# Adipocentric origin of the common cardiometabolic complications of obesity in the young up to the very old: pathophysiology and new therapeutic opportunities

**DOI:** 10.3389/fmed.2024.1365183

**Published:** 2024-04-08

**Authors:** Riccardo Sarzani, Matteo Landolfo, Chiara Di Pentima, Beatrice Ortensi, Paolo Falcioni, Lucia Sabbatini, Adriano Massacesi, Ilaria Rampino, Francesco Spannella, Federico Giulietti

**Affiliations:** ^1^Internal Medicine and Geriatrics, European Society of Hypertension (ESH) “Hypertension Excellence Centre”, Società Italiana per lo Studio dell'Aterosclerosi (SISA) LIPIGEN Centre, IRCCS INRCA, Ancona, Italy; ^2^Centre for Obesity, Department of Clinical and Molecular Sciences, University “Politecnica delle Marche”, Ancona, Italy

**Keywords:** obesity, visceral adiposity, hypertension, type 2 diabetes mellitus, adipocentric, GLP-1 receptor agonists, SGLT2-inhibitors, cardiometabolic complications

## Abstract

Obesity is a multifactorial chronic disease characterized by an excess of adipose tissue, affecting people of all ages. In the last 40 years, the incidence of overweight and obesity almost tripled worldwide. The accumulation of “visceral” adipose tissue increases with aging, leading to several cardio-metabolic consequences: from increased blood pressure to overt arterial hypertension, from insulin-resistance to overt type 2 diabetes mellitus (T2DM), dyslipidemia, chronic kidney disease (CKD), and obstructive sleep apnea. The increasing use of innovative drugs, namely glucagon-like peptide-1 receptor agonists (GLP1-RA) and sodium-glucose cotransporter-2 inhibitors (SGLT2-i), is changing the management of obesity and its related cardiovascular complications significantly. These drugs, first considered only for T2DM treatment, are now used in overweight patients with visceral adiposity or obese patients, as obesity is no longer just a risk factor but a critical condition at the basis of common metabolic, cardiovascular, and renal diseases. An adipocentric vision and approach should become the cornerstone of visceral overweight and obesity integrated management and treatment, reducing and avoiding the onset of obesity-related multiple risk factors and their clinical complications. According to recent progress in basic and clinical research on adiposity, this narrative review aims to contribute to a novel clinical approach focusing on pathophysiological and therapeutic insights.

## Introduction

Obesity is a chronic multifactorial disease characterized by an excessive accumulation of adipose tissue, affecting people of all ages. The World Health Organization (WHO), as well as the World Obesity Federation (WOF) and the World Heart Federation (WHF), defined obesity as a chronic relapsing disease with a body mass index (BMI) ≥ 30 kg/m^2^ (1). Over the last four decades, the global incidence of overweight (BMI ≥ 25 kg/m^2^) and obesity has nearly tripled (2). The WHO predicted that 18% of men and 21% of women will be affected by obesity in 2025, while 40% of the global population will be overweight (3). Recent evidence correlated almost 4 million deaths to obesity and its cardiovascular (CV) complications (4). Life expectancy decreases proportionally with increasing BMI, starting from 25 kg/m^2^ and even more from 27 kg/m^2^ (5). Increased body weight also correlates with a marked increase in healthcare costs. The average annual healthcare cost of an obese adult doubles that of a normal-weight adult in the U.S.A. ($5,010 vs. $2,504) (6).

Genetic, environmental, psychological, socio-economic, and cultural factors contribute to obesity, which may arise at different ages (7, 8). The rare and severe early-onset monogenic forms of obesity led to the identification of major pathophysiological pathways involved in the development of the more common polygenic obesity (9), which is the main focus of this review. The foundation for overweight and obesity development lies in an excessive and disproportionate caloric intake compared to energy expenditure, leading to an accumulation of calories excess in the form of triglycerides stored in the adipocytes that undergo pathological hypertrophy and possibly also hyperplasia (10).

The distribution of excessive adipose tissue is also essential to determine the obesity-related CV and metabolic risk. Individuals with an excessive amount of visceral adipose tissue (VAT), primarily localized intra-abdominally, in the mediastinum, the epicardium, the neck, and even the tongue are at higher CV and metabolic risk than individuals with subcutaneous accumulation of adipose tissue (i.e., in the femoral-gluteal area) (11). Visceral obesity is an independent risk factor for CV disease and mortality (nearly 60% of all CV deaths occur in obese subjects) (4, 12, 13). Indeed, visceral obesity is often associated with several CV and metabolic risk factors, such as arterial hypertension (nocturnal or sustained), metabolic syndrome, insulin resistance, impaired glucose metabolism, type 2 diabetes mellitus (T2DM), dyslipidemia and steatosis/steatohepatitis (4) and their complications, in terms of major CV events, such as acute myocardial infarction and heart failure (often with preserved ejection fraction, HFpEF), atrial fibrillation (AF) and ventricular arrhythmias, stroke, but also chronic kidney disease (CKD), respiratory diseases, such as obstructive sleep apnea syndrome (OSAS), and all-cause mortality (14).

In this regard, we are facing a new era in the pharmacological treatment of obesity and its complications. Among the newer and available drugs, both the glucagon-like peptide-1 receptor agonists (GLP1-RA) and the sodium-glucose cotransporter-2 inhibitors (SGLT2-i) can improve metabolic and CV risk factors by reducing body weight and acting on associated pathophysiological derangements.

This narrative review will range from pathophysiological aspects of obesity, mainly related to excess VAT and its clinical consequences, to the current and future therapeutic approaches of obesity-related CV and metabolic diseases. This paper aims to promote an integrated clinical approach and therapeutic strategy to face the different aspects of obesity-related CV and metabolic complications, beginning from the highly prevalent body overweight, in which the presence of a VAT excess, that we denominate here “visceral overweight,” should never be disregarded, or considered only a mere “amplifier” of CV risk.

## Brief overview of obesity pathophysiology and its cardiovascular and metabolic consequences

We can consider the dietary-polygenic type of obesity as the prototype of a disease in which a polygenic predisposition is coupled with multiple environmental factors, among which an excessive caloric intake with food and beverages coupled with the reduced metabolic rate or, more commonly, the scarce physical activity prevail (15). The genes predisposing to obesity are also widespread in countries with a shortage of food intake; however, this polygenic background helps improving survival without any pathological weight gain. In contrast, whenever food availability is excessive, “emotional” eating and junk food-related gratification can be driven by polygenic traits and are often worsened by a sedentary lifestyle, resulting unavoidably in overweight and obesity.

**Figure 1:**
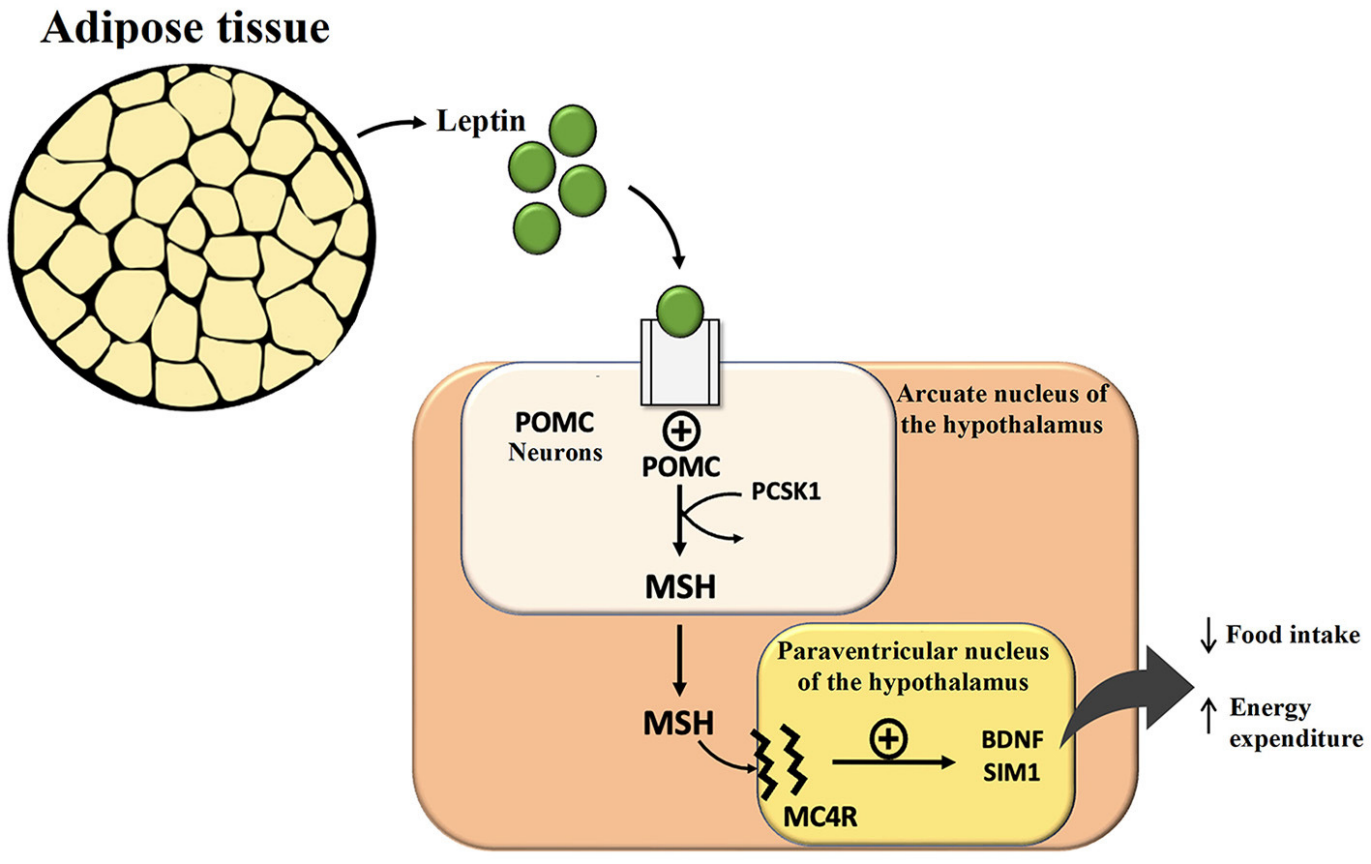
Effect of leptin in the central nervous system and hypothalamic circuits regulating hunger, satiety, and energy expenditure. POMC, pro-opiomelanocortin; PCSK1, proprotein convertase subtilisin/kexin type 1; MSH, Melanocyte Stimulating Hormone; MC4R, melanocortin receptor type 4; BDNF, brain-derived neurotrophic factor.

Monogenic obesity has an early onset in life and severe clinical features (9). Thanks to research on monogenic forms of obesity, derangements in the central nervous system (CNS) pathways have been recognized, especially regarding changes in leptin (LEP) and its receptor (LEPR). Each gene mutation involving these pathways can lead to uncontrolled food intake and obesity development. Leptin is produced by adipose tissue, secondary to its abundance, and is connected to the path centered on melanocortin (MSH, largely α-MSH): hypothalamic neurons in the arcuate nucleus that express pro-opiomelanocortin (POMC) and the agouti-related protein (AGRP) also express LEPR, which binds circulating LEP that reflects the fat amount. POMC neurons, via the MSH component of POMC, interact with neurons that express the melanocortin receptor type 4 (MC4R) in the arcuate and paraventricular nuclei of the hypothalamus, which controls appetite and sense of satiety (Figure 1).

On the contrary, AGRP acts as an antagonist of the MC4R and stimulates increased food intake. Every mutation of genes encoding for these neuronal circuits often simulates leptin deficiency and leads to hyperphagia and severe obesity. Human leptin analogs are available and very effective for treating rare leptin gene mutations and deficiency cases. Setmelanotide, a selective MC4R agonist, has recently been approved by the FDA for the treatment of rare forms of monogenic obesity characterized by LEPR or proprotein convertase subtilisin/kexin type 1 (PCSK1) deficiency, a pivotal enzyme to process POMC into α-MSH that stimulates MC4R (and MC3R) to reduce food intake (9).

Adipose tissue also acts as an endocrine organ affecting the circulating levels of several other hormones: it can secrete not only adipokines (i.e., adiponectin) but also proteins (angiotensinogen) and peptides (angiotensin II), contributing to the overactivation of the renin-angiotensin-aldosterone system (RAAS) and obesity-related hypertension (see below for details) and it can seize and degrade cardiac natriuretic peptides (NPs) by a glucose/insulin-dependent pathway leading to the over-expression of their clearance receptor (NPRC) (16). The lower biological activity of NPs on adipocytes, due to the higher expression of NPRC stimulated by overeating (especially rapidly absorbable sugars), lowers the well-known lipolytic and “browning” activities of NPs, resulting in less beige and brown adipocytes, those rich in mitochondria and ATP-burning heat-producing uncoupling protein 1 (UCP-1), thus contributing to reduced basal metabolism (16, 17). Furthermore, higher VAT is associated with adipocyte hypertrophy, known to be linked with increased insulin resistance, cellular stress and consequent necrosis followed by macrophage recruitment and increased inflammation with the releasing of pro-inflammatory cytokines such as tumor-necrosis-factor alpha (TNFα) and interleukin-6 (IL-6) that contribute to low-grade chronic inflammation and joint obesity-related CV and metabolic complications (10). Adipose tissue in obesity is also associated with capillary rarefaction and reduced delivery of oxygen and nutrients, leading to increased hypoxia-inducible factors and fibrosis (18). Microcirculation dysfunction and rarefaction are hallmarks of arterial hypertension, and some of the findings in obese patients are due to a chronic increase in blood pressure (BP).

Fat accumulation and distribution are also sex- and age-related (19). Excessive subcutaneous fat depots with typical female gluteal and thigh localization usually comprise smaller adipocytes with fewer metabolic abnormalities, identifying a particular phenotype of obese patients, defined as “metabolically healthy obese” (MHO). However, it is an apparent oxymoron nowadays (20, 21). Indeed, MHO patients may already have chronic heart damage (increased interstitial tissue/fibrosis volume and changes in systolic and diastolic functions) predisposing to HFpEF, the most common HF phenotype in the obese population (22) (see below for details). As highlighted in recent literature, the “obesity paradox” described in some clinical contexts, especially in older adults, in whom lean muscle mass is progressively replaced by fat mass, led to the misconception of a possible “*healthy (or even benign)*” obesity. This concept needs to be revised, also through the help of newer anthropometric and metabolic indices more sensitive and specific than BMI, such as the waist-to-height ratio, which has a stronger association with central adiposity-related complications and that should be more frequently used in clinical studies as well as in daily clinical practice (23, 24).

The excess of visceral triglycerides “spillover” can also result in direct lipotoxicity and contribute to chronic organ damage. In the liver, for example, hyperinsulinemia due to visceral obesity-related insulin resistance stimulates the production and accumulation of triglycerides, promoting hepatic steatosis and lipotoxicity, potentially evolving into non-alcoholic steatohepatitis that can progress up to cirrhosis and hepatic cancer (25). These liver changes, also known as non-alcoholic fatty liver disease (NAFLD) and non-alcoholic steatohepatitis (NASH), are considered expressions of metabolic syndrome-related organ damage and are independently associated with increased mortality for CV events (26). A recent “Consensus Statement” involving several scientific societies has proposed a revised nomenclature and classification, defining these alterations as “metabolic dysfunction-associated steatotic liver disease” (MASLD), associating this clinical feature with at least one of the five critical cardiometabolic risk factors (27).

## Obesity, high-normal blood pressure, and arterial hypertension

It is estimated that about three out of four cases of essential arterial hypertension are caused by overweight and obesity (28). Although many genetic and environmental factors contribute to the increase in BP, hypertension is strikingly more prevalent in obese than in normal-weight individuals. In obese hypertensive patients, apart from an excessive dietary salt intake with an average of 10 g/day (29), excessive adipose tissue, especially VAT, is directly involved in the chronic hyperactivation of the sympathetic nervous system (SNS) (30). There is a glomerular hyperfiltration in obesity, which is often coupled with increased tubular sodium reabsorption (31), thus contributing to systemic arterial hypertension and chronic kidney damage. Visceral fat is associated with a “within normal range” increase in plasma aldosterone concentrations, supported by the production of adipokines and angiotensinogen, a fundamental component of the RAAS. This form of “inappropriate secondary hyperaldosteronism” (renin values are inappropriately normal in obese hypertensive despite salt intake and high BP) further favors greater sodium reabsorption, especially at the level of the distal tubule (32). Noteworthy, obese individuals have high serum levels of very low-density lipoprotein (VLDL) cholesterol that induces aldosterone synthesis partly mediated by phospholipase D (PLD) and its enzymatic cascade (33). This obesity-related and VLDL-related alteration of the RAAS, despite the excessive dietary salt intake, maintains mineralocorticoid activity above the overall median values in hypertensive obese patients. Thus, in obese subjects, the aldosterone release and plasma concentrations are set at higher levels than those necessary to maintain BP, leading to inappropriate aldosterone levels (despite they are usually in the “normal” range). In the hypertensive obese, this dysregulation leads to an “escape” from the inhibition of aldosterone secretion during ACE-inhibitors (ACE-I) and AT1 angiotensin receptor blockers (ARBs) therapy (34), which is coupled with an increase in pulse pressure, nighttime BP and cardiac damage (35). In daily clinical practice, the renin-to-aldosterone-ratio (RAR), which is the reciprocal of the ratio that is commonly used to screen for primary aldosteronism (aldosterone-to-renin-ratio, ARR), has been proposed to assess the efficacy of RAAS antagonism, showing the real-life effectiveness of ACE-I or ARBs therapy. A higher RAR was associated with a better 24 h-BP control, especially during night-time (35). Higher RAR values indicate an adequate blockade of the RAAS and, therefore, an adequate therapeutic adherence to ACE-I or ARBs because the increased renin activity is the expression of a reduced negative AT1 feedback on renin secretion together with a lower angiotensin II-dependent aldosterone secretion. On the contrary, lower RAR values can be due to poor adherence or insufficient efficacy/dosage of the drug used, and in the setting of overweight and obese patients highlight a more significant “escape” from the inhibitory action of ACE-I and ARBs, with relative secondary hyperaldosteronism.

Obesity might also lead to hypertension by VAT expansion through mechanical kidney compression and RAAS activation, as visceral perivascular adipose tissue has paracrine effects on the vascular wall, regulating inflammation and RAAS activation (18). Another key pathophysiological mechanism of hypertension development in obese individuals is the impaired blood concentrations, metabolism, and activity of cardiac NPs (ANP and BNP) that exert their activities by binding the NP receptor A (NPRA), which is mainly expressed in the kidney, adipose tissue, adrenal gland, heart, and vascular smooth muscle (32, 36). Both ANP and BNP antagonize the RAAS at multiple levels, decreasing systemic BP but increasing renal perfusion and natriuresis due to a direct proximal tubular inhibition mechanism that leads to macula densa activation followed by afferent arteriole vasodilation and renin secretion inhibition (37). Obese hypertensive patients have reduced plasma NP levels compared to overweight and normal-weight patients. This is primarily due to increased NP clearance mediated by the NPRC overexpression (38, 39), driven by hyperinsulinemia secondary to insulin resistance. Also, the glomerular hyperfiltration in obese subjects (glomerular filtration rate and creatinine clearance increase by 0.7 ml/kg in severe obesity) can lead to an increased renal clearance of NT-proBNP. In addition to NP deficit, other essential determinants of hyperfiltration in obese patients include sodium intake, BP and meat protein consumption. In this context, a new formula for the estimation of creatinine clearance has been proposed in obesity (40).

Conversely, both natriuresis and diuresis associated with fasting-induced BP reduction are thought to result from reduced NPRC expression in adipose tissue (41). Indeed, fasting exerts a specific tissue and gene suppression of the NPRC gene in adipose tissue that seems to be followed by an increase in the biological activity of ANP. In this regard, a drastic decrease in NPRC messenger RNA levels in brown and white adipose tissue has been found, although NPRA expression in adipose tissue is constant or tends to increase (42). Accordingly, ANP infusion was found to have a greater efficacy after a low-calorie diet in obese hypertensive patients (43).

Obese hypertensive patients are, therefore, characterized by a “natriuretic handicap” due to increased degradation and reduced NP biological activity coupled with increased RAAS activity, increased SNS activity and a higher intake of dietary salt. The “swollen feeling,” often reported by obese patients (especially by females), together with leg edemas even in the absence of HF or reduced renal glomerular filtration rate, may be due to the imbalance of sodium and water handling, as well as impaired lipid metabolism, inevitably leading to an increase in BP up to arterial hypertension.

These pathophysiological considerations are also relevant for the HF diagnosis, often a common sequelae of visceral adiposity accumulation and uncontrolled hypertension (44). Since obesity reduces the overall levels of different types of circulating cardiac NPs, even those not taken up by NPRC, the European Society of Cardiology (ESC) has recently proposed different cut-offs for the HF diagnosis in obese patients: a cut-off reduced by 25% when BMI is between 30 and 35 kg/m^2^, a cut-off reduced by 30% when BMI is between 35 and 40 kg/m^2^, and a cut-off reduced by 40% when BMI is over 40 kg/m^2^ (45). These proposed lower cut-offs are being validated in clinical practice.

## Obesity, impaired glucose handling and type 2 diabetes mellitus

One of the most relevant complications related to excessive adiposity is T2DM. Obese patients have a 6.7 times greater risk of developing glucose intolerance and a 4.9 times greater risk of developing T2DM compared to normal-weight patients (46). Despite the well-known T2DM polygenic predisposition, it is estimated that up to 80% of patients would not develop diabetes in the absence of overweight or obesity, which are the primary substrates for insulin resistance and T2DM. In hypertrophic VAT infiltrated by macrophages, inflammatory cytokines (TNFα and IL-6) and reduced adiponectin production [the only well-known “protective adipokine” exerting anti-atherogenic, anti-diabetic, and anti-inflammatory effects (47)] contribute to decreased insulin sensitivity or insulin resistance, leading to dysregulation of glucose handling by the insulin-sensitive tissues. The sedentary lifestyle also contributes to insulin resistance through sarcopenia, leading to a vicious circle that the prediabetic obese generally fails to break, resulting in chronic severe consequences. In clinical practice, overweight/obese patients affected by T2DM were still overweight at the end of several trials based on lifestyle intervention with long follow-up periods, such as the Look AHEAD trial, in which the very modest average weight loss was insufficient to reduce CV outcomes (48). At the same time, a *post-hoc* analysis of the same trial showed that the minority of patients who reached at least 10% weight loss significantly reduced the primary endpoint (49). T2DM in overweight/obese patients is always preceded by a long history of prediabetes, which is often a part of the “metabolic syndrome” driven by insulin resistance, also associated with NAFLD and other well-defined and known metabolic and CV risk conditions. The incidence and prevalence of atherosclerosis and CV events are, therefore, increased even before the diagnosis of overt T2DM (50, 51). The homeostasis model assessment-insulin resistance (HOMA-IR) index, derived from fasting serum glucose and insulin, and the Triglyceride–Glucose (TyG) Index, derived from fasting blood glucose and triglycerides, are the most used indexes to estimate insulin resistance in clinical practice and can help to detect the obese at higher risk of having T2DM (52, 53). Noteworthy, when both familial and polygenic predispositions for T2DM are present, the progression to T2DM is almost unavoidable in this adipocentric clinical setting.

## Obesity and dyslipidemia

**Figure 2:**
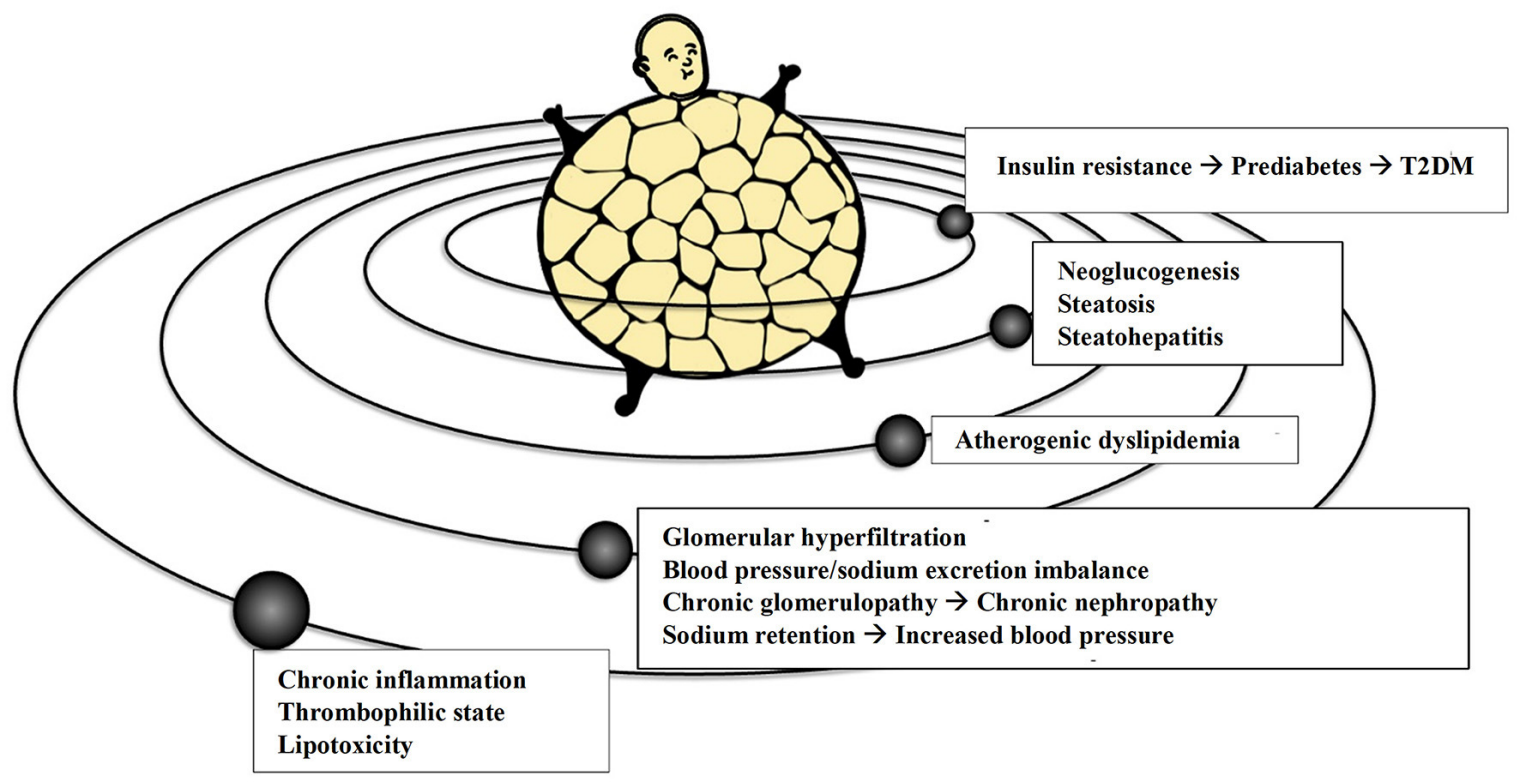
The adipocentric vision of the more common cardio-metabolic and renal conditions. T2DM, Type 2 Diabetes Mellitus.

Obese patients usually have increased circulating VLDL remnants and atherogenic non-high-density lipoprotein (non-HDL) cholesterol. At the same time, high serum triglycerides levels (with also higher postprandial peaks), low circulating HDL levels and increased concentration of the more atherogenic “small and dense” low-density-lipoprotein (LDL) are expected in obesity (54). The enlarged VAT mass exposes the liver, through the portal circulation, to higher levels of free fatty acids (FFA) that impair hepatic lipid and carbohydrate metabolism, especially in the postprandial phase. The increased FFA flux may contribute to the dysregulation of liver metabolism with consequent steatosis and excessive production and secretion of VLDL richer in triglycerides (55). More in-depth, obesity-related hypertriglyceridemia and atherogenic dyslipidemia are the result of induced gene transcription of acyl-coenzyme A hepatic synthetase due to the increasing inflow of FFA to the liver, concurring to hypertriglyceridemia by excessively synthesizing triglycerides, and of the reduced VLDL catabolism in the circulation (56). In addition to these common atherogenic dyslipidemias, visceral overweight and obesity, if concomitant metabolic syndrome or T2DM are also present, can cause severe hypertriglyceridemia with an increased risk of acute pancreatitis, especially in the presence of a predisposing genetic setting and/or excessive intake of carbohydrates, alcohol, and some drugs (57). The consequences mentioned above of abnormal adiposity are graphically summarized in Figure 2.

## Obesity and other relevant cardiometabolic risk factors

*Aging* is characterized by insulin secretion deficiency due to a decreased function of pancreatic beta cells coupled with increased insulin resistance (58). The beta cells' senescence and reduced sensitivity to glucose significantly increase the risk of T2DM. Aging is also associated with ectopic lipid deposition, a pro-inflammatory milieu (“inflammageing”), mitochondrial dysfunction, and accumulation of reactive oxygen species with increased oxidative stress and sarcopenia.

*Sarcopenia* in older obese is particularly relevant because it is associated with reduced production of musclin, a peptide similar to the NPs, but able to selectively block the NPRC, thus increasing NPs levels and their positive biological activities. Notably, the sarcopenic reduction of musclin facilitates the onset of HF (37). Furthermore, older obese often suffer from osteoarticular diseases, thus contributing to even lower levels of physical activity.

*Cigarette smoking*, a CV risk multiplier, causes pro-atherogenic and inflammatory damage. In daily clinical practice, the fixed-dose combination of naltrexone and bupropion, a drug combination approved for weight management, could be helpful in obese smokers because of the positive effects of bupropion on tobacco dependence symptoms after withdrawal. However, it is essential to note that bupropion therapy requires proper monitoring of arterial BP, given its slight hypertensive effect (59).

*Obstructive sleep apnea syndrome (OSAS)* increases the risk of CV events, mainly stroke. While sleeping, upper airways collapse with subsequent intermittent hypoxia and hypercapnia (60). Such alterations determine the activation of the SNS, endothelial system dysfunction and reduction of the physiological nocturnal BP dip, contributing at first to nocturnal hypertension and then to sustained and drug-resistant hypertension (61). OSAS is a widespread condition in obesity, contributing to the onset and progression of CV risk factors and diseases (62).

*Peripheral arterial disease (PAD)* is a highly prevalent condition whenever obesity is complicated with T2DM and arterial hypertension. Macrovascular and microvascular arterial damage (both atherosclerosis and arteriosclerosis) can be easily detected in retinal, cerebral, cardiac, lower limbs and other arterial districts (63, 64). Documented PAD is equivalent to previous CV events such as myocardial infarction, predisposing to further new ones and therefore requiring a reduction of LDL cholesterol below 55 mg/dL or even below 40 mg/dL using combinations of proven to be effective lipid-lowering drugs (statins, ezetimibe, bempedoic acid, PCSK9 inhibitors, and inclisiran).

*Dementia* development is favored by obesity together with aging, mainly because of its link with higher BP and T2DM. Moreover, adipokines secreted by the excess adipose tissue significantly impact the CNS by increasing leptin resistance (65) and insulin resistance (66). A recent study confirmed the association of brain aging with common CV risk factors, including excessive body weight (67). The adequate control of the obesity-related risk factors to prevent irreversible cerebral consequences should be a priority.

## Global cardiovascular risk assessment in obesity

**Figure 3:**
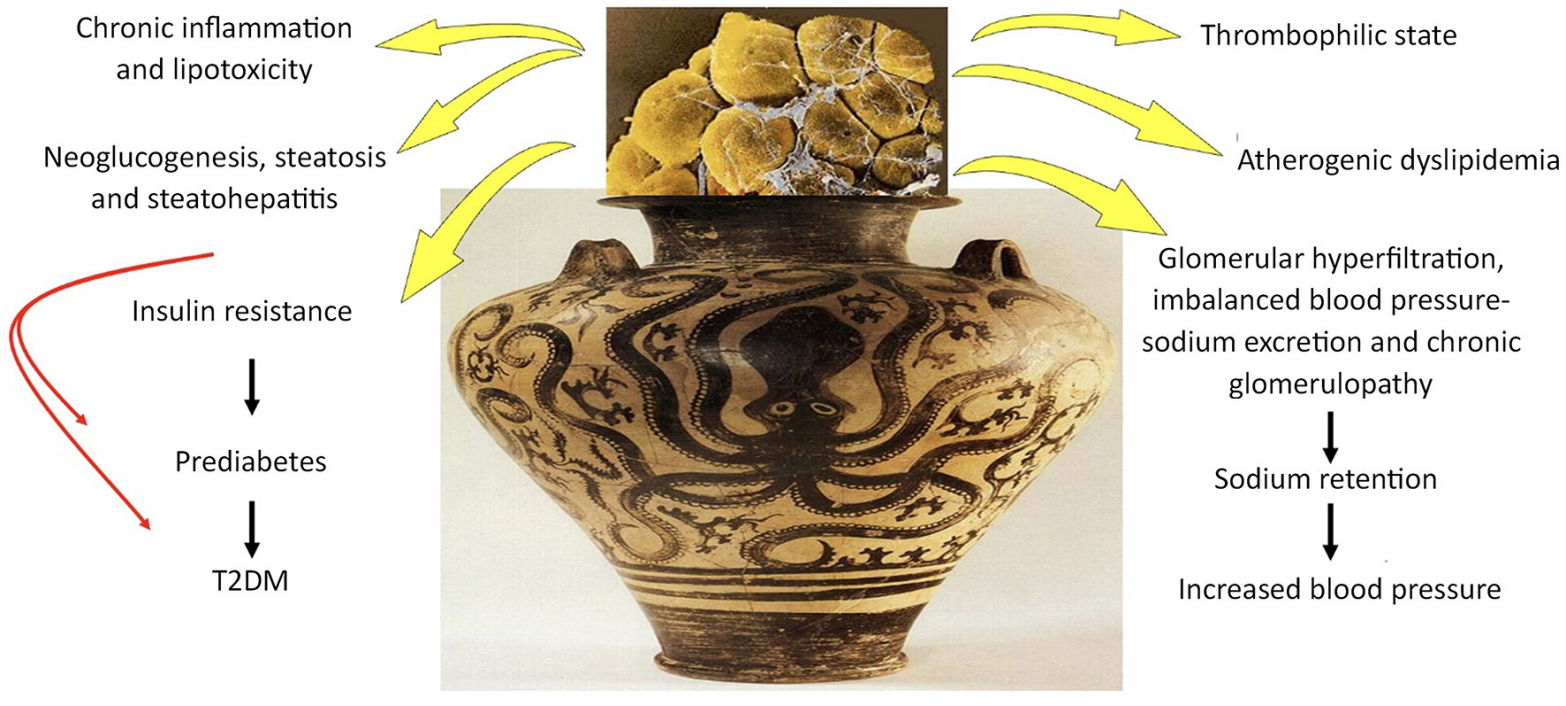
Visceral obesity: the Pandora's Jar of the twenty-first century. T2DM, Type 2 Diabetes Mellitus.

An individual global CV risk assessment is mandatory for overweight and obese patients. However, the evaluation of the overall CV risk in the obese using the European Society of Cardiology (ESC) endorsed Systematic Coronary Risk Estimation (SCORE2 and its counterpart for older patients SCORE2-OP) models and charts (68) is still not widely used in daily clinical practice. Therefore, easy-to-use multilanguage web-apps (e.g., www.humtelemed.it) have been developed to help less experienced physicians to assess CV risk adequately. Furthermore, a specific tool has recently been developed in diabetic patients (the SCORE2-diabetes), that allows a more accurate assessment of the CV risk in this population (69). Apart from using these models and charts in primary prevention, assessing CV risk in a patient with visceral obesity could be pretty complex. Considering all the adiposity-related complications reminds the opening of a modern version of the mythical “Pandora's Jar” (Figure 3), which at present, among the non-transmissible diseases, mainly contains visceral obesity and its CV and metabolic complications, such as increased insulin resistance and T2DM, atherogenic dyslipidemia, altered natriuresis with increased BP and arterial hypertension, glomerular hyperfiltration leading to CKD, increased gluconeogenesis, steatosis and hepatic lipotoxicity with steatohepatitis, and ultimately major CV events and CV death.

## Evaluation of obesity in clinical practice: low-cost clinical tools to improve phenotyping

Estimating VAT amount can unmask normal-weight individuals (according to BMI) who may have metabolic syndrome and, therefore, be classified as metabolically obese normal-weight patients. On the contrary, the so-called MHO phenotype meets the BMI values for obesity, but does not have the clinical features of metabolic syndrome, not even insulin resistance. Although it is not a benign condition, MHO individuals usually have larger fat storage capacity in subcutaneous tissue, lower visceral and ectopic fat levels, less liver steatosis, and a lower degree of systemic inflammation (70). Whereas, short-term cross-sectional studies suggest that MHO men and women are not at increased risk of CV disease, longitudinal studies indicate that this phenotype may not be benign and that this group is at higher risk for increased carotid artery intima-media thickness, coronary calcification, impaired vasoreactivity, and other CV events, as well as all-cause mortality (71). However, they do not usually manifest associated comorbidities, such as prediabetes, dyslipidemia, or hypertension. Therefore, clinicians should view MHO as a transient or intermediary state that, in many cases, may progress over time to an evident unhealthy obesity phenotype. Reduced cardiorespiratory fitness might be related to MHO. Cardiorespiratory fitness lowers the risk of all-cause mortality for metabolically unhealthy individuals with obesity and those with and without the MHO phenotype, suggesting that including the evaluation of cardiorespiratory fitness along with BMI and waist circumference may improve the assessment of risk status (10). Recent evidence further downgraded the benign nature of MHO, showing increased extracellular components and myocardial fibrosis with subclinical alterations of both systolic and diastolic function, the seeds of the development of HFpEF (22).

Several methods are available for evaluating and phenotyping obesity and its associated cardiometabolic risk. The distribution and amount of adipose tissue can accurately be assessed using imaging such as magnetic resonance (MRI) and computed tomography (CT) that represent the “gold standards.” However, these ideal methods of VAT evaluations in overweight and obesity are usually performed only for research purposes in small groups of selected patients. Non-invasive methods, such as bioimpedentiometry, dual energy X-ray absorptiometry (DEXA) or total body 3D scanners, are useful to identify body fat percentage and distribution (72, 73), although even these tools are not widely available and cost-efficient on large scales. Overall, the quality of anthropometric measurements is of fundamental importance. Nowadays, waist circumference (WC) is still the most used method to estimate visceral obesity (74, 75). The waist-to-hip ratio is another traditional tool to describe visceral obesity (76), whereas extensive recent studies showed that the waist-to-height ratio should be preferred (23, 77). Indeed, an “obesity survival paradox” has been reported in HF patients, especially in older adults: obese subjects might have better outcomes and lower mortality risk compared with normal weight in the setting of HFrEF. However, this spurious result was not confirmed when the data were adjusted for waist-to-height ratio. Both BMI and waist-to-height ratio showed that greater adiposity is associated with a higher risk of the primary outcome and HF hospitalization, with more robust evidence for waist-to-height ratio (24). In addition, the association between BMI (and potentially any other anthropometric index) and outcome in patients with HFrEF is also confounded by the relationship between adiposity and NPs levels, which are typically lower in obesity (45). BMI should be replaced in patients with HFrEF and daily clinical practice because alternative anthropometric measurements showed a more precise association between greater adiposity and a higher risk of HF hospitalization. Regardless of the anthropometric index, greater adiposity was associated with worse symptoms and health-related quality of life (24). These measurements could also demonstrate a link between adiposity and mortality in the older population (78).

The frequent association between visceral adiposity, hepatic steatosis and dyslipidemia with increased circulating triglycerides due to increased synthesis and/or reduced catabolism of VLDL introduced the so-called “hypertriglyceridemic waist” phenotype, in which the increased abdominal circumference (90 cm in men and 85 cm in women, lower values than the standard criteria for the diagnosis of metabolic syndrome) is coupled with hypertriglyceridemia (2 mmol/L or 177 mg/dl in men and 1.5 mmol/L or 133 mg/dl in women). The hypertriglyceridemic waist phenotype is binary (presence or absence), so it cannot be used as a continuous variable to evaluate its correlation with visceral adipose tissue; in any case, the presence of hypertriglyceridemic waist phenotype is associated with a 75–85% probability of increased amount of visceral adipose tissue (79, 80).

Using fasting triglyceridemia (TG) and glycemia in the TyG index, a reliable insulin-resistance and metabolic syndrome marker and an independent predictor of CV events has been tested (81, 82). TyG index is calculated with the following formula: ln [fasting triglycerides (mg/dl) fasting plasma glucose (mg/dl)/2]. TyG index below 4.5 is considered normal in both men and women. TG-to-HDL-C ratio is also used in clinical practice. Recent studies showed that an increased TG-to-HDL-C ratio is associated with a higher prevalence of pre-hypertension and arterial hypertension, even in normoglycemic individuals, despite the TyG index turning out to be more accurate. High levels of both these indices strongly correlate with an increased global CV risk, and this association was partly mediated by increased prevalence of dyslipidemia, T2DM, and hypertension (81). The metabolic score for insulin resistance (METS-IR) estimates the cardiometabolic risk in healthy and at-risk subjects, representing a promising screening for insulin resistance. It is calculated with the following formula: ln [2 FPG (fasting plasma glucose) (mg/dl) + fasting TG (mg/dl)] BMI (kg/m^2^)/ln [HDL-C (mg/dl)]. Recent studies showed that the METS-IR score might be a valid indicator of overall CV risk. It may predict the risk of coronary calcification (and therefore the CV risk) in asymptomatic patients without obvious CV pathologies (83, 84).

In addition to waist-to-height ratio, other indexes that take into consideration both anthropometric and metabolic parameters together, have been validated and disseminated in clinical practice. These tools are easily applicable and useful to identify a “functional” excess of visceral adiposity even in overweight patients. The visceral adiposity index (VAI) has been recently developed. It is an empirical-mathematical gender-specific model based on simple anthropometric (BMI and WC) and functional parameters (TG and HDL-C) indicative of fat distribution and function. It was found to be a marker of cardiometabolic risk and has a strong independent association with CV and cerebrovascular events (85). Another helpful tool is the lipid accumulation product (LAP) index calculated from WC and TG, which strongly indicates insulin resistance, metabolic syndrome, T2DM, and NAFLD (86). Recently, a novel score (METS-VF) was developed to estimate VAT by combining the METS-IR, waist-to-height ratio, age and sex, showing a better performance compared to other VAT surrogate formulas and the ability to predict incident T2DM and hypertension (87).

In conclusion, these low-cost bio-anthropometric indexes should be applied more widely in clinical practice, especially to characterize overweight or obese non-diabetic individuals with a high prevalence of metabolic syndrome, prediabetes, pre-hypertension and arterial hypertension. The improvement in phenotyping will allow the detection of patients at higher metabolic and CV risk, thus needing more focused and intense treatment. Clinicians should become more confident with adiposity evaluation through these usual and novel anthropometric measurements and formulas. An adipocentric-guided clinical evaluation should focus more on a broader use of these indexes.

## Cardiometabolic effects of weight loss

Weight loss in obesity is linked to a multitude of positive consequences on CV and metabolic health. Losing weight improves insulin resistance, which is completely reversible in the early stages, thus preventing the development of T2DM. More considerable weight loss can also lead to T2DM vanishing. It also reduces systolic BP by at least one mmHg per kg of weight loss (88). In many cases, especially in younger individuals with high-normal BP or stage 1 arterial hypertension, normotension can still be achieved by only losing weight (49). It is important to remember that an average reduction of 2 mmHg in the medium-long term corresponds to a 10% risk reduction of stroke and a 7% risk reduction of ischemic heart disease (89, 90), stressing the role of weight loss as a therapeutic strategy to reduce BP and CV disease burden. Cardiac overload is also reduced by weight loss, thus avoiding or slowing the development and progression of HF. Weight loss also improves sleep quality by reducing the severity of OSAS, leading to improvement in night-time BP profiles. Weight loss reduces circulating lipids, especially non-HDL cholesterol and triglycerides. These improvements in circulating lipids can also lead to a reduction in liver steatosis. All the above changes benefit CV and renal health, reducing glomerular hyperfiltration and albuminuria and slowing down the reduction of kidney function (91).

## Treatment of visceral overweight and obesity: an adipose-related approach between established therapies and newest pharmacological interventions

Lifestyle changes, including nutrition/behavioral interventions, have been and still are the first step in the clinical management of overweight and obesity (92). However, it means not only more physical activity and diet, but also acting concretely on the individual familial and social background to build a supportive environment allowing the patient to maintain a healthy lifestyle as long as possible (55). Early education at school, population-based messages about physical activity, healthy foods and early care, and worksite health promotion programs are all valuable strategies still not efficaciously implemented in many countries (70). In the overweight or obese older subjects, diet and lifestyle should modulate weight loss to avoid losing muscle mass. Unless CKD is present, a caloric deficit of ~500 kcal/day with at least 1,000–1,200 kcal/day with 1gr/Kg of protein has been suggested (93). Nevertheless, many studies showed a substantial failure of the several proposed diets and lifestyle approaches in the long term (years) because individuals tend to regain all the weight and adiposity lost. This is particularly true outside the clinical trials, where the patients are usually closely followed, given that in real life, worldwide overweight and obesity have not only been reduced by decades of predicated lifestyle changes but are instead increasing steadily in both incidence and prevalence.

Lifestyle approaches to treat obesity have been recently summarized in several guidelines, and we did not address this topic in this conceptual review. Instead, we will focus on effective surgical and pharmacological treatments that are now available, making lifestyle changes more effective and long-lasting.

In treated overweight and obese patients with VAT, beyond the percentage weight loss, the novel cardiometabolic indexes of visceral “functional” adiposity, such as the above mentioned LAP, VAI or METS-VT, can be easily monitored in clinical practice and could be good efficacy parameters to take into consideration. Given the global effects of weight loss, even the clinical indexes, such as accurate BP measurements or glycated hemoglobin, can be used as well.

## Bariatric surgery

We chose to present the relevance of bariatric surgery first because of its conceptual significance, given that it proved the critical role played by excessive caloric intake and adiposity in determining T2DM, dyslipidemias, hypertension, and CV diseases and, on the other hand, the beneficial effects of weight loss linked to surgery. Bariatric surgery is a therapeutic option generally reserved for severe obesity who failed non-pharmacological and pharmacological approaches. It has been, so far, the only therapeutic strategy that proved to be effective for the most severe forms of obesity, leading to significant weight loss with improvement or even remission of its related complications. The most used procedures in bariatric surgery are sleeve gastrectomy, gastric bypass and intragastric balloon, which are indicated especially in class II and III obese after accurate clinical assessment, including psychological aspects. Arterial hypertension, T2DM and dyslipidemia generally improve significantly or can even resolve after bariatric surgery (94). All the baseline anthropometric parameters, such as body weight, BMI, WC, and abdominal excessive adipose tissue measurements significantly improved 3 months after surgery. A decrease in extra-abdominal adipose tissue (especially the cardiac one) was also documented. Moreover, bariatric surgery led to more remarkable percentage changes in all anthropometric “adipose” measurements compared to diet and exercise (95). Despite its demonstrated benefits in patients with severe obesity, bariatric surgery is still an underused therapeutic option performed only in a small number of selected severely obese patients because of its costs and possible complications related to surgical procedures (3).

## Pharmacological treatment of obesity

There are several medications approved and available for clinical use in overweight and obese patients. The fixed-dose combination of phentermine/topiramate was not approved for clinical use in the European Union. These drugs are described below, from the older ones in availability to the incoming ones.

*Orlistat* is a selective inhibitor of pancreatic lipase. It reduces dietary fats' absorption, resulting in increased fecal excretion. A moderately low-calorie and a lower-fat diet should always be combined. It is indicated for the treatment of obese patients with a BMI > 30 kg/m^2^ or overweight patients with a BMI > 28 kg/m^2^ and associated risk factors and has been approved at a dose of 120 mg three times daily by both EMA and FDA (also as over-the-counter drug). The prevalent bowel side effects strongly reduce the adherence and persistence to treatment. Indeed, the daily losses of fatty, orange-colored and malodorous liquid stools usually create discomfort on the one hand. On the other hand, however, it can be used as an “index” of the dietary fat intake, thus encouraging adherence to a low-calorie diet with reduced fat content to decrease the amount of fat excretion and the burden of side effects.

*Bupropion/Naltrexone* is a fixed-dose drug combination approved for obesity treatment. Bupropion is a dopamine and norepinephrine reuptake inhibitor used effectively for the treatment of depression and for tobacco smoking cessation (59). Naltrexone is an opioid receptor antagonist used to treat alcohol and opiate dependence. Pro-opiomelanocortin (POMC) neurons are powerfully stimulated by dopamine and norepinephrine. This combination would determine a dopamine-mediated stimulation of the hypothalamus, thanks to the reuptake inhibition due to bupropion, to produce more POMC and more MSH, leading to greater MC4R activation, with decreased appetite and increased satiety. Specifically, naltrexone would reduce the effects of beta-endorphin by blocking opioid receptors that inhibit POMC secretion, diminishing the desire and the search for certain foods and the consequent pleasure in consuming them. This combination was approved in the early 2010's by EMA and FDA and is indicated, in addition to a low-calorie diet and physical activity, for weight management in adult patients with an initial BMI of 30 kg/m^2^ (96). It could also be used starting from a BMI of 27–30 kg/m^2^ in the presence of at least one weight-related comorbidity, such as prediabetes or T2DM, or when GLP1-RA are not tolerated. However, there is no data regarding CV safety (no CV outcome studies published), and bupropion can increase both BP and heart rate (97, 98). From phase III studies, the combination of bupropion/naltrexone appeared to increase systolic and diastolic BP of up to 3 mmHg compared to placebo (99) due to the increase in norepinephrine caused by bupropion. However, BP levels would appear to decrease after 56 weeks of persistence on treatment. In any case, any BP reduction linked to weight loss was absent. A 4–6% reduction in body weight compared to placebo was only obtained at the maximum dosage, with common side effects such as nausea, headache, and constipation (97, 98). Therefore, this association is not indicated in obese with high-normal BP or hypertension. Otherwise, if the subject is on effective antihypertensive therapy and other obesity treatment cannot be tolerated, this combination can be considered to lose weight.

*Liraglutide* is still the only GLP1-RA approved for the treatment of weight loss in most countries. In patients not affected by T2DM, the drug is generally not reimbursed by national health systems, such as in Italy. At the dose of 3 mg/day, it is indicated in adult patients with BMI ≥ 30 kg/m^2^ or in adult patients with BMI ≥ 27 kg/m^2^ in the presence of at least one weight-related comorbidity, such as prediabetes or T2DM, hypertension, dyslipidemia or OSAS. In one of the SCALE studies, performed in non-diabetic patients without any drugs that could significantly impact weight loss/gain (obesity and prediabetes study), liraglutide 3 mg reduced weight in 63% of the subjects during 56 weeks, with 14.4% of patients that lost 15% of their weight, starting from an average BMI of 38.3 kg/m^2^ (100, 101). Weight loss was mainly mediated by reduced appetite and calorie intake rather than increased energy expenditure and was accompanied by a significant reduction in BMI and abdominal circumference. A decrease in both systolic and diastolic BP, blood glucose concentration, VLDL and triglycerides, and PCR has been observed, while on the contrary, adiponectin increased as expected (101, 102).

The side effects of liraglutide, mainly consisting of moderate nausea, vomiting or diarrhea, are generally mild, usually dose-dependent, transient and self-limiting, and this is the reason why this drug should always be started at lower doses. Severe adverse events occurred in 6.2% of patients treated with liraglutide and in 5% of patients treated with placebo, events unrelated to the drug. Gallbladder stones and cholecystitis occurred with a slightly higher frequency in the liraglutide group, and this side effect was noticed in several other weight loss trials (103, 104), as they are linked to weight loss itself in the obese and not so much to the drug (105). Indeed, the lower food intake and weight loss may lead to altered production and bile secretion coupled with a reduction in gallbladder emptying that might contribute to increased symptomatic gallstones in patients treated with liraglutide (106).

*Semaglutide* was found to be very effective in reducing body weight in non-diabetic obese patients taking 2.4 mg of the drug in the STEP studies. STEP-1 study enrolled a total of 1961 obese non-diabetic adult patients with a BMI > 30 kg/m^2^ or a BMI > 27 kg/m^2^ coupled with at least one obesity-related comorbidity (hypertension, dyslipidemia, CV disease, obstructive sleep apnea), showing an average decrease of 15.3% in body weight in the group treated for 68 weeks with 2.4 mg (starting from 0.25 mg and then titrated) of semaglutide associated with a healthy lifestyle, compared to 2.6% body weight loss in the placebo group (107, 108). In addition, the STEP-1 study showed a net decrease in inflammatory markers, BP, blood lipids and glycosylated hemoglobin. The STEP 3 study enrolled 611 patients always treated with 2.4 mg of semaglutide (titrated from 0.25 mg) in association with a targeted behavioral therapy, showing a 17.6% weight loss compared to a 5% weight loss in the placebo group (109). These data were subsequently confirmed by the STEP-4 study that enrolled 902 patients, demonstrating an average reduction of 14.8% in body weight in the treated group. Moreover, this study found a marked average decrease in the abdominal circumference (−9.7 cm) and systolic arterial BP (−3.9 mmHg) coupled with an improvement in the physical function score (secondary endpoints). Despite gastrointestinal side effects in 49% of the study population, only 2.4% of treated patients stopped the drug (110). During the following years, several studies confirmed a weight loss between 7 and 11%. In addition, semaglutide has been compared in recent studies with liraglutide in non-diabetic obese, showing that the association with a healthy diet and a healthy lifestyle leads to a more marked reduction in body weight than liraglutide. In the recently published STEP-HFpEF trial, 529 obese (mean BMI 37 kg/m^2^) hypertensive (82%) non-diabetic older adults (mean age 69 years) with HFpEF were randomized to semaglutide 2.4 mg vs. placebo for 52 weeks (111). The primary composite endpoint, a better Kansas City Cardiomyopathy Questionnaire score and a significant weight loss (-13.3%), was obtained with semaglutide compared to placebo (112). Furthermore, an improved 6-min walking distance and a 21% reduction in NT-proBNP levels confirmed that excessive weight plays a crucial role in HFpEF and the reduction of hemodynamic load also by a significant BP decrease (−4.9 mmHg) may be of great benefit in these patients. Moreover, semaglutide showed the ability to improve NASH, with no worsening of fibrosis in NASH patients, leading to reduced serum transaminase, liver fat content and liver stiffness (113, 114).

The SELECT trial, a landmark study in paving the way for a new real-life adipocentric approach to CV disease, enrolled 17,604 adults aged 45 years or older with overweight or obesity and established CV disease with no prior history of diabetes mellitus and showed a significant reduction (−20%) in major CV events (MACE) in patients taking semaglutide 2.4 mg vs. placebo. The study's primary endpoint was defined as the composite outcome of the first occurrence of MACE (defined as CV death, non-fatal myocardial infarction or stroke). The three primary endpoint components contributed to the MACE reduction (115).

Semaglutide is also available for oral administration once daily through the development of a particular technology that involves the co-formulation of semaglutide with an absorption booster, the sodium salcaprozate [Sodium N-(8-(2-hydroxybenzyl-Amino) caprylate] (SNAC). In the PIONEER-6 trial (116), oral semaglutide was found to be not inferior, but not superior to placebo, in the primary CV outcome, and a further study (SOUL trial) on 9,642 subjects aiming at proving the effectiveness of oral semaglutide compared to placebo in reducing the CV outcome is currently ongoing (117). Real-world data is emerging on its safety and efficacy in lowering glycated hemoglobin, BMI, and BP (118). Oral semaglutide at 14 mg showed a similar BP lowering of 2 mmHg as subcutaneous semaglutide at 1 mg, an amount of reduction that can already justify a reduction in CV events and mortality (119). Also, after 6 months of treatment, it reduced fatty liver index and VAT, preserving fat-free and skeletal muscle mass, evaluated with bioimpedentiometry (120). Thanks to their weight loss properties, which involve all the body's adipose tissue deposits (119), GLP1-RA improves all CV risk factors, such as BP and lipid profile (121). A novel, potent preventive approach to CV disease will be based on a substantial weight loss strategy achievable only when a GLP1-RA is associated with lifestyle modifications.

*Tirzepatide* is a dual GLP1 and GIP (glucose-dependent insulinotropic peptide, formerly called gastric inhibitory peptide) receptor agonist recently approved for obesity treatment in both the U.S.A. and Europe. Regarding pharmacodynamics, tirzepatide couples the activation of the GLP1-RA to the effect on the GIP receptor, which is responsible for most of the action in stimulating insulin secretion (122). The phase III SURMOUNT-1 trial studied the efficacy and safety profile of 10 or 15 mg tirzepatide in patients not affected by T2DM treated for 72 weeks, showing a significant decrease in both body weight and abdominal circumference. Indeed, after 24 weeks of treatment with the maximum dosage of the drug, it led to a 17% average reduction of the abdominal circumference, a 34% reduction of the total fat mass, a 0.5 points average reduction of glycosylated hemoglobin, a 28% average reduction of circulating triglycerides and an 8-mmHg average reduction of systolic BP (123). In addition, more than 95% of the patients who entered the study with prediabetes had normoglycemia by the end of the study. These data are highly significant to demonstrate further how excessive calorie intake and adiposity are primary factors in determining the leading CV and metabolic complications of overweight and obesity, especially prediabetes/T2DM and hypertension, that may resolve with an appropriate weight loss. Given its five-day half-life, tirzepatide is administered subcutaneously once a week, as well as semaglutide. Similarly, side effects (mainly nausea, diarrhea, and constipation) were modest and transient and occurred with increasing doses. Patients on tirzepatide were found to have a higher prevalence of cholecystitis than patients on a placebo. Still, this evidence appears to be related to obesity itself and the lower food intake coupled with a more rapid weight loss in obese patients rather than a direct effect of the drug (124). The results of the SURPASS-CVOT trial are expected in 2024 (125). This randomized, double-blind phase III study is enrolling participants with T2DM and atherosclerotic CV disease. It aims at evaluating the CV outcomes of the therapy with tirzepatide by assessing the non-inferiority and superiority of this drug compared to the GLP1-RA dulaglutide. The expectations for this new molecule are high, and the scientific community worldwide already speaks of a revolutionary drug from the metabolic point of view, especially for its superior effectiveness in inducing weight loss. Another CV outcome-focused trial in obese patients not affected by T2DM should end in 2027 (SURMOUNT MMO), evaluating the effect of tirzepatide on morbidity and mortality reduction in established CV disease. In the meantime, the weight loss found in the SURMOUNT-3 and SURMOUNT-4 trials in a combined total of 1,249 randomized adults adds positive results to the data previously reported from more than 3,400 patients randomized in SURMOUNT-1 and SURMOUNT-2 trials. In the SURMOUNT-3 trial, tirzepatide titration to a 10-/15 mg weekly dose, coupled with intensive lifestyle management, showed a 21% average reduction in body weight after 72 weeks from randomization, compared to a 3.3% average increase in body weight among controls (126). The 94% of patients on tirzepatide achieved at least a 5% weight loss. The SURMOUNT-4 assessed a tirzepatide discontinuation, highlighting that those who received tirzepatide continuously for 88 weeks (the 36-week run-in phase plus the 52-week randomized phase) had an overall 26% average weight loss from baseline. Conversely, the group that switched to placebo had an average weight gain of 14.8% (127). Among the predictive factors for a ≥15% weight loss, there were higher tirzepatide dose, female sex, white or Asian race, younger age, undergoing treatment with metformin, better glycemic control (based on lower glycated hemoglobin and lower fasting serum glucose) and lower non-HDL cholesterol level (128). In T2DM patients treated with basal insulin, tirzepatide showed a better lowering of glycated hemoglobin and fewer hypoglycemia episodes than prandial insulin (129), proving an excellent alternative. The findings from this trial pointed out the highest level of weight loss observed in the SURMOUNT program and the importance of ongoing tirzepatide treatment to maintain weight loss.

It is evident, summarizing the results obtained from these novel drug therapies for obesity, that these can be addressed as real “adipocentric” drugs able to reduce any metabolic and CV risk factor along with weight and visceral adiposity. In this perspective, today's primary goal is to raise awareness of the adipocentric nature of the CV and metabolic diseases affecting overweight and obese patients, aiming at setting up an effective combined non-pharmacological treatment coupled with the substantial and unprecedented help of these novel pharmacological weapons. At this point, clinicians should not only treat the obesity-related serious consequences but should aim at preventing them, mainly through early and intensive management of overweight and obesity. Effective treatment of overweight and obesity with the new adipocentric drugs, primarily directed toward those with higher CV risk in which a “multi-specialist” approach and treatment would be necessary, will lead to better patient health and lower global cost burden.

*Other incretin mimetics*, such as the once-daily oral non-peptide GLP1-RA orforglipron tested in phase II trials in patients with and without T2DM, are under investigation. It reported a reduction of up to 2.1% of glycated hemoglobin and a decrease in mean body weight at week 26 of up to 10.1 kg in T2DM patients (130). In comparison, a weight reduction of at least 10% by week 36 was observed in up to 75% of patients at a dose of 45 mg in non-diabetic obese patients with related improvement in their cardiometabolic measures (131). Two dual agonists of both GLP-1 and glucagon receptors, pemvidutide (NCT05295875) and survodutide (NCT06077864, NCT06066515), produced significant weight loss and other cardiometabolic benefits in preliminary randomized trials and are in development, especially for the treatment of obesity and NASH (132, 133). Lastly, retatrutide is supported by phase 2 trials. It is a triple G agonist, a single peptide that activates GLP-1, GIP, and glucagon receptors. Thanks to the activation of the glucagon receptor with its catabolic effects (adipose lipolysis, food intake reduction, slower gastric emptying, and increased energy expenditure), while the two systems counterbalance the hyperglycemic effect, it managed to achieve a 24% weight loss over 48 weeks at the highest dose (12 mg) in obese patients without T2DM (134). This weight loss was associated with improved BP and glycated hemoglobin levels, fasting glucose, insulin, total and LDL cholesterol and triglycerides. However, 16% of subjects have discontinued the drug at the highest dose for adverse events, mainly due to nausea and non-serious gastrointestinal effects (134, 135). The main drugs available to treat obesity and their main clinical characteristics are summarized in Table 1.

**Table 1:** Main characteristics of the pharmacological treatments against obesity.

**Drug treatment**	**Clinical indications**	**Mechanisms of action**	**Beneficial effects**	**Side effects**
**Orlistat** 120 mg tid	BMI >30 kg/m^2^ or overweight patients with a BMI >28 kg/m^2^ and associated risk factors combined with lifestyle modification (low-fat and low-calorie diet regimen, physical activity).	Selective inhibition of pancreatic lipase, reduction of dietary fats absorption (up to about 30%).	Limited weight loss with scarce improvement in CV risk and events.	Abdominal discomfort: increased and uncontrolled bowel movements, abdominal pain, greasy and malodorous stools, intermittent loss of oil or liquid feces (“spotting”)
**Naltrexone/bupropion** 7.2/78 mg up to qid	BMI of 30 kg/m^2^ or BMI of 27–30 kg/m^2^ in the presence of at least one weight-related comorbidity, such as prediabetes or T2DM combined with lifestyle modification (low-fat and low-calorie diet regimen, physical activity).	Dopamine-mediated stimulation of hypothalamic production of POMC and MSH and block of opioid receptors that inhibit POMC secretion leading to decreased appetite, increased satiety, and less tendency to food craving.	Limited weight loss with scarce improvement in CV risk and events.	•Common side effects such as nausea, headache, and constipation. •Increased blood pressure and heart rate. •Careful administration in hypertensives (only if another treatment approach is not tolerated or contraindicated).
**Liraglutide** 0.6–3 mg die (sc) **Semaglutide** 3–14 mg die (oral) 0.25–2.4 mg/week (sc)	BMI ≥30 kg/m^2^ or in adult patients with BMI ≥27 kg/m^2^ in the presence of at least one weight-related comorbidity such as prediabetes or T2DM, hypertension, dyslipidemia or OSAS combined with lifestyle modification (low-fat and low-calorie diet regimen, physical activity).	•Reduction of appetite and food ingestion by activating GLP-1 receptors in the hypothalamus. •Full-stomach sensation due to slower gastric emptying. •Better glycemic control by activating GLP-1 receptors in the pancreas, enhancing insulin and reducing glucagon release in a glucose-dependent manner.	•Enhanced postprandial and fasting glycemic control, improved pancreatic beta-cell function and possibly preserved beta-cell mass. •Subcutaneous injection formulation of liraglutide and semaglutide demonstrated a favorable effect on blood pressure, lipid profile, CV risk, MACEs, CV mortality, and heart failure incidence independently of diabetic status, along with a substantial weight loss and abdominal circumference reduction. •Oral semaglutide reduced weight and visceral adiposity. Clinical trials to assess its potential CV benefits in the non-diabetic obese are ongoing.	Most reported side effects (mainly nausea, diarrhea, and constipation) are modest and transient and occur with increasing doses. Less common but more severe complications are related to pancreatic and biliary tract inflammation cases.
**Tirzepatide** 2.5–15 mg/week (sc)	BMI ≥30 kg/m^2^ or in adult patients with BMI ≥27 kg/m^2^ in the presence of at least one weight-related comorbidity such as prediabetes or T2DM, hypertension, dyslipidemia or OSAS combined with lifestyle modification (low-fat and low-calorie diet regimen, physical activity).	Dual GLP1 and GIP receptor agonist. Mechanisms of action are similar to those of GLP1 receptor agonists.	•Enhanced postprandial and fasting glycemic control, improved pancreatic beta-cell function and possibly preserved beta-cell mass. •Substantial weight and abdominal circumference reduction. •Favorable effect on blood pressure and lipid profile independently of diabetic status. •Demonstrated CV safety. •Based on the premises of single GLP1 receptor agonists, trials focused on evaluating the efficacy of reducing the incidence of CV outcomes are ongoing.	Most reported side effects (mainly nausea, diarrhea, and constipation) are modest and transient and occur with increasing doses. Less common but more severe complications are related to pancreatic and biliary tract inflammation cases.

BMI, body mass index; CV, cardiovascular; GLP-1, glucagon-like peptide 1; GIP, glucose-dependent insulinotropic peptide; T2DM, type 2 diabetes mellitus; OSAS, obstructive sleep apnea syndrome; POMC, pro-opiomelanocortin; MSH, melanocyte-stimulating hormone; CNS, central nervous system; MACE, major adverse cardiovascular events; sc, subcutaneous administration.

Overall, the changes in anthropometric and cardiometabolic parameters in obese patients under pharmacological treatment are usually variable. This well-known variability can depend on genetic or social factors, but it likely mainly depends on a large variability in diet and lifestyle adherence. The novel pharmaceutical approaches, especially those based on GLP1-RA if well-tollerated can reduce such variability by improving adherence to diet. Current evidence on the new anti-obesity drugs found a magnitude of weight loss almost comparable to bariatric surgery in some cases, varying on the basis of the molecule used, with a weight loss plateau after 12–18 months (136). However, their benefits partly regress after withdrawal, as demonstrated by the SURMOUNT-4 trial (127), supporting the hypothesis of the need for their chronic use. This is also the reason for a need of a guided lifestyle approach that should be undertaken in these patients, together with the introduction of the anti-obesity drug, in order to maintain weight loss in the long term (136). Real-life studies on large populations and of longer duration will be able to provide us with further information in the coming years.

## Pharmacological treatment of obese patients affected by T2DM

Recently published guidelines went into detail regarding the critical importance of excessive adiposity in the genesis and management of most cases of T2DM. The first essential therapeutic intervention is a low-carbohydrate and low-calorie diet subdivided into smaller meals and coupled with an increase in physical activity. Still, new drugs are already a valuable resource for managing obese T2DM patients with promising and lasting results. Treatments of obesity associated with T2DM could be effectively evaluated and managed according to the most recent internationally published guidelines (69, 137, 138). Therefore, in this conceptual review, we will deal only with some pivotal concepts that are still often disregarded even in the specialistic setting. Beyond glycemic control, the new therapies are focused on CV disease and CKD prevention.

In managing obese patients affected by T2DM, the GLP1-RAs have recently gained a key spot, given their effectiveness in weight reduction. In 2020, the American Association of Clinical Endocrinologists and American College of Endocrinology (AACE/ACE) Consensus Statement had already recommended the use of GLP1-RAs and SGLT2-i independently of glycaemic control and as the first choice, especially in the presence of atherosclerotic CV disease, in patients at high risk of CV events, in patients with stage 3–4 CKD, and patients affected by HFrEF (139). The Kidney Disease Improving Global Outcomes (KDIGO) 2022 Guidelines in patients affected by T2DM and CKD with an estimated glomerular filtration rate (eGFR) between 60 and 30 ml/min and/or albuminuria recommended SGLT2-i as the first choice, and GLP1-RA in addition as second choice (140). The most recent 2023 ESC Guidelines on Cardiovascular Disease Prevention in patients with T2DM strongly advised first obtaining adequate weight loss using GLP1-RA (69).

Any reduction of ventricular wall stress, like that resulting from weight loss and the consequent decrease in blood volume and BP during GLP1-RA treatment, is also accompanied by a reduction in circulating NT-proBNP levels, as a measure of a reduced NPs secretion from the heart (141) because there is a very rapid resetting of cardiac NPs secretion and their clearance. Moreover, GLP1-RA, thanks to the weight loss, indirectly facilitates sodium elimination by improving NPs action through lower food intake and then reduced expression of NPRC that is insulin/glucose dependent in adipocytes (16).

Benefits of weight loss, mainly due to reduced caloric intake, have already been described above for semaglutide and liraglutide (142–144). Liraglutide treatment in patients with T2DM was accompanied by lower CV mortality and lower all-cause mortality in the landmarking LEADER study (144). Several studies on semaglutide in T2DM (SUSTAIN 1-5) demonstrated its effectiveness at 0.5 and 1 mg daily doses in reducing body weight and glycated hemoglobin (142, 145–148). The SUSTAIN-6 CV outcome study showed a 26% reduction in the overall risk of death from CV causes, non-fatal acute myocardial infarction, or non-fatal stroke (149). The significant decrease in body weight, BP and glycated hemoglobin accompanied a 36% reduction in the risk of new nephropathy or renal function worsening, resulting in excellent metabolic, CV and renal benefits (150).

Another GLP1-RA approved for T2DM therapy is dulaglutide, which has a homology of about 90% with native human GLP1 and resistance to degradation by the enzyme dipeptidyl peptidase IV (DPP-IV) and large dimensions that slow its absorption and reduce its clearance at the renal level. A soluble formulation with a half-life of 4.7 days is administered once a week subcutaneously. The long-term study on CV outcomes (REWIND) (151) recruited a different T2DM population than previous CV outcome studies with GLP1-RA. Indeed, only 31.5% of the study participants had a history of CV disease known at the time of recruitment. Most patients presented only CV risk factors or subclinical vascular damage and were in primary prevention. The dulaglutide group maintained better glycemic control and lower levels of some CV risk factors compared to placebo throughout the follow-up. A possible more significant effect on stroke was observed compared to non-fatal myocardial infarction, and the nephroprotective effect was confirmed (151). Similarly, the dual GLP1 and GIP receptor agonist, tirzepatide, will be essential in managing patients with obesity and T2DM. Many patients taking tirzepatide lost 10% or more of their body weight, a threshold value effectively associated with greater metabolic and CV benefits. Other GLP1-RA agonists, dual (GLP-1 and GIP) and triple (GLP-1, GIP and glucagon) receptor agonists, are also in an advanced phase of clinical development and testing in clinical trials and will be available soon (134, 152).

**Figure 4:**
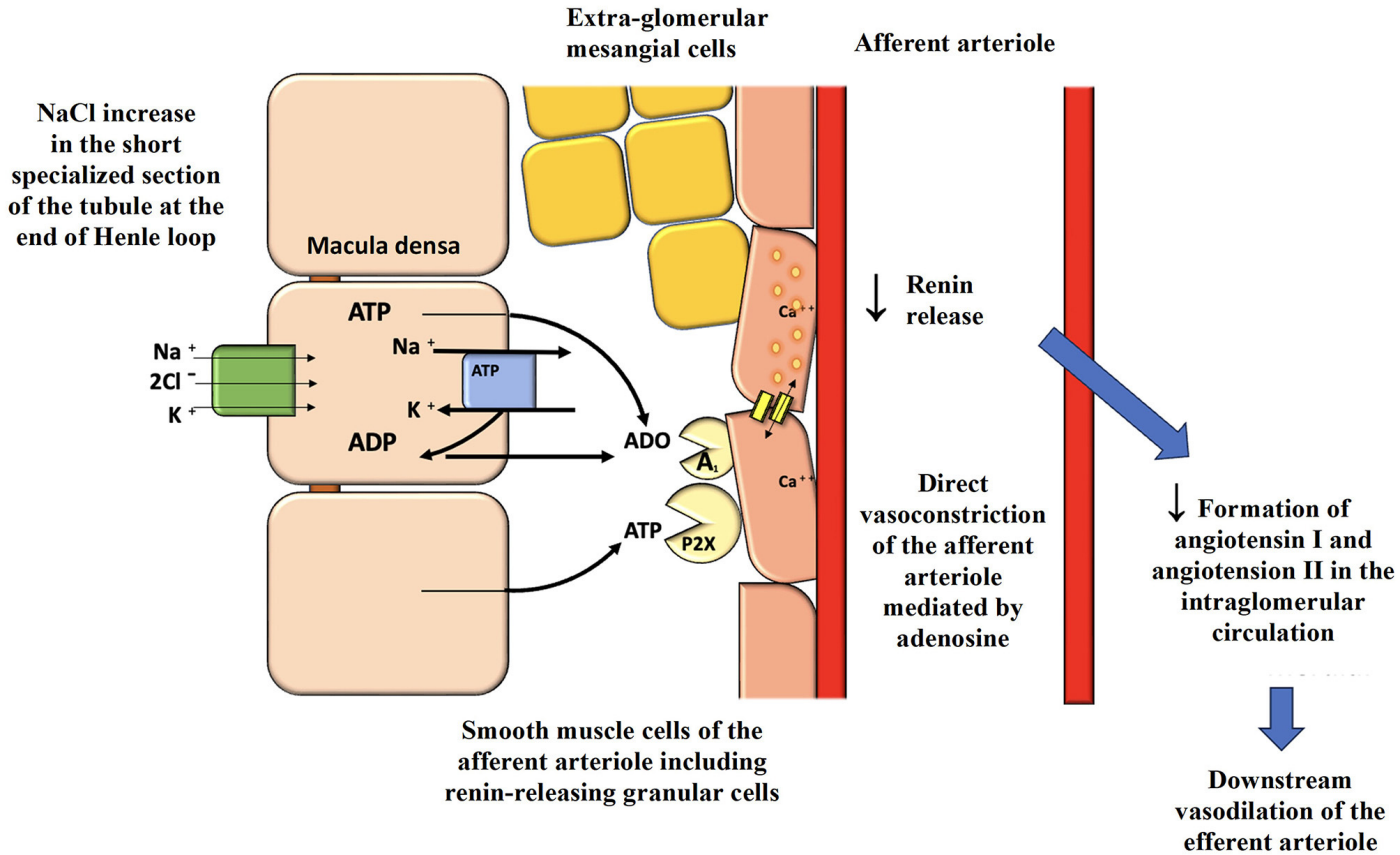
Activation of the macula densa by SGLT2 inhibitors and consequences on glomerular hemodynamics and renal protection. SGLT2, sodium-glucose cotransporter-2; ATP, adenosine triphosphate; ADP, adenosine diphosphate; ADO, adenosine.

Other key classes of molecules for the treatment of obese patients with T2DM are SGLT2-i (empagliflozin, dapagliflozin, canagliflozin, ertugliflozin, and sotagliflozin), “hybrid” and “dual” diuretics able to inhibit the RAAS, unlike the other available and “classic” diuretics (153). They act on the proximal tubule of the nephron, promoting natriuresis and glycosuria with osmotic diuresis. The inhibition of the SGLT2 cotransporter leads to reduced proximal reabsorption of glucose and sodium chloride, just as NPs do. The sensor of the macula densa, the Na/K/2Cl cotransporter, perceives an increased amount of sodium (but especially senses chlorine), interpreting this as glomerular hyperfiltration and/or increased sodium chloride in the body. This results in an increased release of the nucleotides ATP and ADP from the macula, which rapidly catabolize to the nucleoside adenosine at the vascular pole of the glomerulus of the same nephron, with stimulation of type 1 adenosine receptors and subsequent direct vasoconstriction of the afferent arteriole together with a reduced release of renin from the granular smooth muscle cells of the same afferent arteriole (Figure 4). Importantly, this will also lead to lower local production of angiotensin I and lower arrival of angiotensin II to the efferent arteriole, which is rapidly produced by the local ACE present throughout the glomerular endothelium, resulting in less vasoconstriction of the efferent arteriole and lower glomerular hyperfiltration in obese. The term “double” diuretic applied to SGLT2-i is due to the natriuretic and osmotic diuretic effects. The term “hybrid” is related precisely to the ability of this molecule to reduce RAAS activity, behaving in the opposite way to loop diuretics (that block the macula densa sensor) or distal convolute tubule thiazide diuretics (153). Moreover, SGLT2 blockade is linked to inhibition of the Na-H-exchanger NHE3 with further natriuresis (154). These characteristics also determine their effectiveness in reducing BP by lowering distal tubule sodium reabsorption due to the lower renin activity. In comparison, ARBs and ACE inhibitors, especially at the low dosages commonly used in clinical practice, cannot completely counteract the “intra-nephron” effects of locally produced angiotensin II, which can also be produced by renin-formed angiotensin I via alternative routes to ACE. In addition, ARBs and ACE inhibitors stimulate renin secretion from the afferent arteriole because they hinder inhibiting its secretion via the angiotensin II-dependent negative feedback. Noteworthy, in obese patients, an “escape” from the effects of ACE inhibitors or ARBs on the control of aldosterone secretion can be observed, possibly also due to the simultaneous enhanced renin activity (34). The ability of SGLT2-i to reduce plasma volume and body sodium without stimulating the intrarenal RAAS, except for an increase in system RAAS in an early acute phase due to the rapid lowering of plasma volume, with consequent BP lowering, produces a reduction of cardiac afterload. Due to the consequences of SGLT2 cotransporter inhibition, there will be significant cardio- and renal-protective benefits that have revolutionized therapies in these fields irrespective of the presence of T2DM (155). In addition, this drug class leads the patient to lose about 70 grams of glucose per day (280 kcal), facilitating weight loss in overweight or obese patients with a documented reduction of VAT. Dapagliflozin facilitates an average weight loss of up to a maximum of 6.4% and reduces glycated hemoglobin by 1%, allowing an average reduction of at least 18 IU of insulin. In the DECLARE study, in which diabetic patients were also obese (mean BMI 32.1 kg/m^2^), no increased risk of severe hypoglycemia was observed with dapagliflozin therapy compared with placebo. Thanks to its features, dapagliflozin led to a reduction in HF hospitalizations and mortality in patients with HF regardless of ejection fraction (156). Several observational data indicated how SGLT2-i decreased the sodium concentration in interstitial fluid with a consequent interstitial fluid clearance without changing the intravascular volume by osmotic diuresis (157). Dapagliflozin slowed renal function decline over the years by up to 28% in diabetic patients with diabetic nephropathy. The DELIVER study also found its benefits in patients with HF and preserved ejection fraction (158). In the DAPA CKD trial, dapagliflozin was found to reduce the risk of end-stage renal disease (ESRD) by 50%, also reducing the risk of progression of renal damage and death from renal or CV causes, when used in patients with CKD regardless of the presence or absence of DM (159). The same outcomes were also found for empagliflozin regarding HF patients with preserved and reduced ejection fraction and patients with CKD (160–162). Regarding Canagliflozin, the CANVAS study, conducted in T2DM patients with CV disease or high CV risk, aimed to demonstrate the safety and CV efficacy of the drug. The results showed a reduction in CV events in patients treated with canagliflozin compared to the placebo group but an increased risk of metatarsal or toe amputation (163). In the CREDENCE trial, canagliflozin was given to T2DM patients with CKD, reducing renal and CV events (163, 164). Using canagliflozin, as expected based on the class effects of SGLT2-i, resulted in an initial drop of eGFR with subsequent stabilization and longer-term renal protection. At the same time, canagliflozin was very effective in lowering systolic BP, thus reducing the need for further medications to lower BP in already treated hypertensive patients (165). These three gliflozins have demonstrated very similar and very positive metabolic, CV, and renal effects, even in patients not affected by T2DM, but only empagliflozin and dapagliflozin to date have the clinical indication for CKD and HF with reduced or preserved ejection fraction, regardless the presence of T2DM. The safety of such therapies is not a particular concern, and to limit their most common predictable side effects (urinary tract infections), patients are only requested to stay well-hydrated and maintain good genital hygiene after each urination.

## Pharmacological treatment of obese patients affected by arterial hypertension

Overweight or obese patients who are also affected by arterial hypertension usually need antihypertensive combination strategies to obtain proper 24-h coverage (90, 166). First-choice drugs are ARBs or ACE-I, preferably used at the highest dosages (167). For example, in the DROP study (168) on hypertensive obese patients with T2DM and microalbuminuria, the most effective dosage of valsartan, both for BP control and for microalbuminuria reduction, was 640 mg per day (considering the maximum available dosage in a single pill is 320 mg). Irbesartan has obtained excellent dosage-dependent results regarding nephroprotection in diabetic patients with microalbuminuria. The IRMA-2 study on 590 hypertensive patients with T2DM showed that irbesartan can prevent progression to overt nephropathy (169). The IDNT study, conducted on 1,715 hypertensive with T2DM and proteinuria with advanced stages of nephropathy, showed that irbesartan at 300 mg, compared with amlodipine, can protect against the risk of ESRD or death (170). The REIN study, conducted with ACE-I in chronic but non-diabetic nephrotic patients, showed unequivocally that the renal protective effect of ramipril was absent in people with normal BMI and was instead maximum in obese people both for progression of renal damage and for proteinuria (171).

In most of the obese patients, to obtain an adequate BP control, diuretic therapy is usually needed. The combination of ACE-I or ARBs with thiazide or thiazide-like diuretics down to about an eGFR of 30 ml/min/1,73 m^2^ or the use of loop diuretics (torasemide is preferable over furosemide due to its longer half-life and its higher bioavailability after oral intake) when eGFR <30 ml/min/1,73 m^2^ is useful and often necessary to control BP in obese hypertensives (166, 172). Diuretics help excrete the excess dietary sodium, whereas ARBs or ACE-I reduce the unwanted effects of the consequent diuretic-mediated RAAS stimulation (172). In clinical practice, to increase treatment adherence, single-pill fixed-dose triple combinations of ARB or ACE-I plus a thiazide diuretic (or the thiazide-like indapamide) and amlodipine are available, such as two combinations containing an ACE-I (perindopril or ramipril) and two combinations containing an ARB (olmesartan or valsartan). Amlodipine blocks the L-type calcium channels found mainly in the glomerular afferent arteriole and, unfortunately, could favor glomerular hyperfiltration in obese patients. Antagonists of the RAAS should always counterbalance the action of diuretics or direct vasodilators such as amlodipine. Otherwise, there could be an increase in diuresis with nocturia, reduced nephroprotective effect, and postural oedema. An amlodipine-based monotherapy could lead to significant peripheral oedema due to a vasodilating direct impact, so an association with a RAAS blocker should be preferred to reduce this unpleasant but avoidable effect (173). Lercanidipine can be used as an alternative to amlodipine, for a more balanced dilation of the efferent arterioles due to L-type and T-type calcium channel blockade. The latest version of the European Society of Hypertension (ESH) Guidelines emphasized how single-pill double or triple-fixed-dose combinations with a diuretic can resolve the efficacy problem frequently found in obese hypertensives, thus increasing therapeutic adherence (90).

Beta-1 selective or super-selective adrenergic blockers such as bisoprolol or nebivolol can help maintain the heart rate <70 bpm in sedentary obese patients. Beta-1 blockers help reduce renin secretion after beta-1 adrenergic stimulation by sympathetic nerves on the renin-secreting cells of the afferent glomerular arteriole.

Obese patients affected by arterial hypertension may also need a mineralocorticoid receptor antagonist (MRA; e.g., canrenone) to obtain adequate control of 24-h BP due to the above-explained “escape” phenomenon and the persistent inappropriate secretion of aldosterone despite the use of ACE-I or ARBs in the overweight/obese subjects (34). For an effective and complete 24-h BP control, these subjects, especially if with low RAR values, are the best candidates for MRA or the “new” aldosterone synthase inhibitor (174, 175) (i.e., baxdrostat and lorundrostat). Classic MRAs, such as canrenone, are usually considered as a fourth choice in obese hypertensive patients uncontrolled by a triple therapy with an ACE-I or ARB, a thiazide (or thiazide-like) diuretic and a calcium channel blocker (CCB) (90). Recent studies on finerenone (a non-steroidal antagonist of the mineralocorticoid receptor) found that it can reduce and maintain systolic BP 3 mmHg lower approximately, and, likely, even more on 24 h or at night (not evaluated in published trials). It can reduce both renal and CV damage in patients with diabetic nephropathy who are usually obese, lowering the risk of kidney damage and HF hospitalization (176). This novel aldosterone antagonist, studied for target organ protection, might be particularly valuable in obesity-related hypertension.

The association of antihypertensive therapy (ACE-I or ARB plus a CCB) with an SGLT2-i has broad implications, together or in alternative to “classical” diuretic treatment, in reducing BP and increasing at the same time the cardiorenal protection with clinical indications regardless the presence of T2DM. In this setting, a recent substudy of the SURMOUNT-1 (177) was conducted on 494 overweight and obese (BMI ≥ 27 Kg/m^2^) non-diabetic hypertensive patients to assess the impact of different doses of tirzepatide on BP profiles evaluated with ambulatory BP monitoring (ABPM). After 36 weeks, reductions in 24-h systolic and diastolic BP were dose-dependent and consistent across subgroups of participants stratified by clinically relevant confounders, including age, sex, BMI, and hypertension-related factors. This study demonstrates that tirzepatide improves 24-h BP in obesity-related hypertension. Even night-time systolic BP, which is often poorly controlled in overweight and obese patients, being a stronger predictor of CV and all-cause death than daytime and 24-h systolic BP, was also significantly reduced by tirzepatide. Furthermore, correlation and mediation analyses indicated that these effects of tirzepatide on BP are independent of weight loss, therefore, adding a GLP1-RA, or a double GLP1/GIP-RA, in overweight/obese non-diabetic hypertensive patients could be the ultimate fundamental improvement in BP management and relative target organ protection.

## General considerations on drug therapy for cardiometabolic prevention in the obese patient

Evidence-based therapies for CV risk reduction are still not adequately implemented in clinical practice, even in obese patients affected by T2DM with proven CV damage. In a recent study of Italian specialized centers on diabetes care, only 26.8% of T2DM patients at high CV risk were treated with high-intensity statin therapy, only 3.9% with GLP1-RA and only 2.8% with SGLT2-i. It was a fact that only 3.6% of patients requiring all three treatment classes received such prescriptions, while 42.6% received no prescriptions (178). Beyond and regardless of obesity, there is still too much therapeutic inertia to the prescription of drugs that have proved to be safe and effective in reducing mortality from CV events (56). For example, statins proved to be safe and preventive even in patients affected by NAFLD and NASH, reducing the risk of both cirrhosis and hepatocellular carcinoma in obese subjects (179).

An adiposity-centered approach to overweight and obesity will open a new era in the treatment of the main consequences of chronic excessive adiposity, such as arterial hypertension, T2DM, CKD, and HF. The two recently introduced therapies, GLP1-RA and SGLT2-i, are fundamental in the management of obese patients, especially in the presence of multiple CV risk factors and/or cardio-renal damage. We believe that overweight patients defined by BMI criteria need to improve their diet and lifestyle first, but if they suffer from comorbidities, such as metabolic syndrome, prediabetes and hypertension and are not able to lose weight or ameliorate their risk profile through diet and lifestyle alone, anti-obesity drugs, especially GLP1-RA, can already have a role in therapy. The robust evidence mentioned in our review showed that losing adiposity with the help of GLP1-RA can ameliorate all the cardiometabolic risk factors, corroborating the standard therapies for hypertension, dyslipidemia and diabetes thus improving the chances to reach therapeutic goals more easily. Until a few years ago, it was challenging for a patient affected by obesity and T2DM to reach back a condition of euglycemia by losing 10–20% of his body weight, also improving dyslipidemia, hypertension and OSAS. This condition was steadily achieved only with bariatric surgery, keeping in mind all the costs and the possible related complications. This novel pharmacological approach based on these drugs, previously limited to T2DM patients only, opens new perspectives. The use of these drugs leading to weight loss and exerting diuretic action may also help simplify the burden of the antihypertensive therapy, leading to a more considerable reduction of CV risk at the same time. In addition, the use of these drugs in the early stages of metabolic impairments, such as in prediabetes and/or metabolic syndrome, where insulin resistance is present without overt organ damage yet, will even prevent the progression to diabetes and chronic kidney and heart damage. Indeed, GLP1-RA and SGLT2-i in the early phases counteract renal hyperfiltration and the consequent glomerular sclerosis and/or microalbuminuria with progressive loss of nephrons as well as chronic BP-related conditions leading to macro- and micro-vascular arterial damage, cardiac and cerebral ischemic events, and HFpEF. CV outcome studies have confirmed the efficacy of both GLP1-RA and SGLT2-i in reducing CV and renal events and mortality in obese with or without T2DM.

Nowadays, in many countries, such as Italy, among the GLP1-RA, only liraglutide is indicated (but not reimbursed by the national health system) in non-diabetic obese patients with high CV risk. However, semaglutide is likely to get the approval for the same indication, with the advantage of a single weekly administration. On the other hand, SGLT2-i are indicated and reimbursed not only in T2DM patients but also in HF patients with reduced or preserved EF, as well as in CKD patients, regardless of the presence of T2DM.

## Conclusion

**Figure 5:**
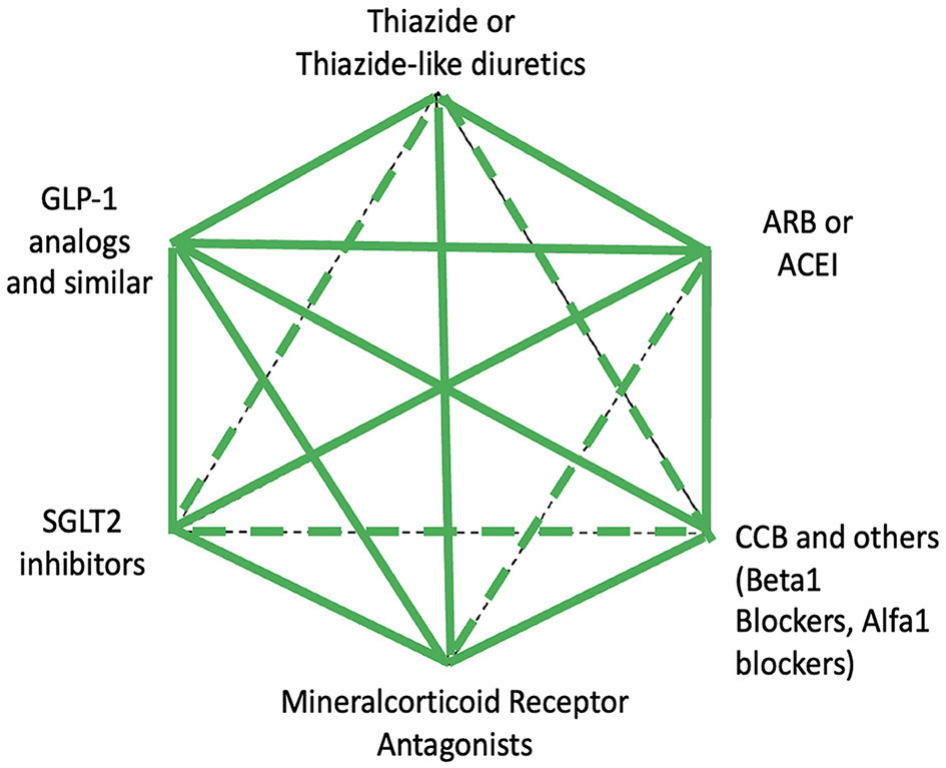
Possible drug associations for the hypertensive obese patient with or without impaired glucose metabolism and/or cardio-renal organ damage. The continuous lines indicate proved safe and effective associations whereas intermittent lines indicate less tested or less indicated associations. GLP-1, glucagon like peptide-1; SGLT2, sodium-glucose cotransporter-2; ARB, angiotensin receptor blocker; ACEI, angiotensin-converting enzyme inhibitors; CCB, calcium channel blocker.

The pathophysiological and clinical concept of “adipocentrism” in managing obesity-related conditions should lead to a therapeutic strategy to reduce body weight and improve BP, lipids, and glucose levels. It should be planned as soon as possible, applying what we named “adipocentric approach.” After decades of lifestyle-only interventions as an “ancillary” treatment that often failed to reach the desired outcomes, especially in the medium and long term, the availability of innovative drugs is likely to change radically the management of obesity and its related complications. In the light of this novel therapeutic era, for the obese affected by arterial hypertension as a practical example, we propose an adipocentric modification of the historical figure of Irvine Page by introducing novel therapeutic combinations for the treatment of obese hypertensive patients, also considering the expected associations with T2DM, CKD, and HF (Figure 5).

Soon, the combination of GLP1-RA and SGLT2-i is likely to become the first choice for the treatment of obese patients, even with high-normal BP and prediabetes (and/or metabolic syndrome). In obese patients affected by arterial hypertension, adding a GLP1-RA and an SGLT2-i on top of a RAAS antagonist and the often-needed diuretic can provide effective metabolic, CV, and renal protection, especially in the presence of subclinical or clinical target organ damage. Furthermore, lipid-lowering treatments for primary and secondary CV prevention should be frequently associated, keeping in mind that the use of the novel obesity-centered therapeutic strategies in the background is likely to facilitate the achievement of the therapeutic goals for LDL values (i.e., LDL cholesterol below 70 or below 55 mg/dL in high and very high-risk patients, respectively) with less pharmacological intensity (lower dosages and/or lower number of lipid-lowering drugs needed).

In conclusion, an adipocentric approach aiming at treating early and intensively, especially the VAT excess, rather than waiting for the dangerous adiposity-related CV and metabolic comorbidities to develop, may facilitate a more effective reduction of CV, cerebrovascular, and renal events in most overweight and obese patients. Global healthcare costs are also likely to be consistently reduced.

## Author contributions

RS: Conceptualization, Data curation, Funding acquisition, Investigation, Methodology, Project administration, Resources, Supervision, Validation, Visualization, Writing – original draft, Writing – review & editing. ML: Data curation, Formal analysis, Investigation, Software, Supervision, Writing – original draft, Writing – review & editing. CD: Data curation, Investigation, Supervision, Visualization, Writing – original draft, Writing – review & editing. BO: Data curation, Investigation, Writing – original draft, Writing – review & editing. PF: Data curation, Investigation, Writing – original draft, Writing – review & editing. LS: Data curation, Investigation, Writing – original draft, Writing – review & editing. AM: Data curation, Investigation, Writing – original draft, Writing – review & editing. IR: Data curation, Investigation, Writing – original draft, Writing – review & editing. FS: Conceptualization, Data curation, Formal analysis, Investigation, Methodology, Software, Supervision, Validation, Visualization, Writing – original draft, Writing – review & editing. FG: Conceptualization, Data curation, Investigation, Methodology, Project administration, Supervision, Validation, Visualization, Writing – original draft, Writing – review & editing.
